# Morphology Dependence Degradation of Electro- and Magnetoactive Poly(3-hydroxybutyrate-co-hydroxyvalerate) for Tissue Engineering Applications

**DOI:** 10.3390/polym12040953

**Published:** 2020-04-20

**Authors:** Luis Amaro, Daniela M. Correia, Pedro M. Martins, Gabriela Botelho, Sónia A. C. Carabineiro, Clarisse Ribeiro, Senentxu Lanceros-Mendez

**Affiliations:** 1 Center of Physics, Universidade do Minho, 4710-057 Braga, Portugal; luisamaromartins@gmail.com (L.A.); d.correia@fisica.uminho.pt (D.M.C.); pamartins@fisica.uminho.pt (P.M.M.); 2 Center of Chemistry, Universidade de Trás-os-Montes e Alto Douro, 5001-801 Vila Real, Portugal; 3 Department of Chemistry, Universidade do Minho, 4710-057 Braga, Portugal; gbotelho@quimica.uminho.pt; 4 LAQV-REQUIMTE, Department of Chemistry, NOVA School of Science and Technology, Universidade NOVA de Lisboa, 2829-516 Caparica, Portugal; sonia.carabineiro@fct.unl.pt; 5 CEB—Centre of Biological Engineering, University of Minho, 4710-057 Braga, Portugal; 6 BCMaterials, Basque Center for Materials, Applications and Nanostructures, UPV/EHU Science Park, 48940 Leioa, Spain; lanceros@fisica.uminho.pt; 7 IKERBASQUE, Basque Foundation for Science, 48013 Bilbao, Spain

**Keywords:** piezoelectric materials, poly(hydroxybutyrate-co-hydroxyvalerate), hydrolytic degradation, tissue engineering

## Abstract

Poly(hydroxybutyrate-co-hydroxyvalerate) (PHBV) is a piezoelectric biodegradable and biocompatible polymer suitable for tissue engineering applications. The incorporation of magnetostrictive cobalt ferrites (CFO) into PHBV matrix enables the production of magnetically responsive composites, which proved to be effective in the differentiation of a variety of cells and tissues. In this work, PHBV and PHBV with CFO nanoparticles were produced in the form of films, fibers and porous scaffolds and subjected to an experimental program allowing to evaluate the degradation process under biological conditions for a period up to 8 weeks. The morphology, physical, chemical and thermal properties were evaluated, together with the weight loss of the samples during the in vitro degradation assays. No major changes in the mentioned properties were found, thus proving its applicability for tissue engineering applications. Degradation was apparent from week 4 and onwards, leading to the conclusion that the degradation ratio of the material is suitable for a large range of tissue engineering applications. Further, it was found that the degradation of the samples maintain the biocompatibility of the materials for the pristine polymer, but can lead to cytotoxic effects when the magnetic CFO nanoparticles are exposed, being therefore needed, for magnetoactive applications, to substitute them by biocompatible ferrites, such as an iron oxide (Fe_3_O_4_).

## 1. Introduction

Polymeric materials with different morphologies are processed as scaffolds to mimic the extracellular matrix (ECM) and to manipulate cell response and engineer specific tissues [1]. Thus, a proper biomimetic strategy must allow the scaffold to mimic the native tissue environment, including morphological features, conditions and stimuli, to stimulate cells in a specific differentiation path [2,3,4].

In particular, smart polymers are gaining increasing attention as substrates and scaffolds for tissue engineering applications mainly as electroactive substrates—mostly piezoelectric ones—such as poly(l-lactic acid) (PLLA) [5,6], poly(hydroxybutyrate) (PHB) [7], poly(hydroxybutyrate-co-hydroxyvalerate) (PHBV) [8,9] and poly(vinylidene fluoride) (PVDF) [10,11], among others. The ability of those materials to actively enhance and stimulate cellular differentiation processes has been already proven [12,13], based on their mechano-transduction characteristics, generating voltage upon mechanical stimulation and vice-versa [14]. Piezoelectricity is a property that appears in a diversity of human tissues, including DNA, bones or tendons, emphasizing the relevance of electrical and mechano-electrical stimulation in physiological processes [15,16]. In a different approach to apply mechanical and/or mechano-electrical signals, magnetoelectric materials have also proven their aptness for tissue engineering applications, with the particularity of allowing electrical stimulation of the materials and, therefore, on the cells cultured on them, through magnetic solicitation [17,18]. These materials can generate voltage upon magnetic stimulation through the coupling of the magnetostrictive effect (magnetic to mechanical) and piezoelectric effect (mechanical to electric) [19,20]. Magnetoelectric composites are thus achieved combining a piezoelectric polymer with magnetostrictive particles [21,22,23], and their potential for tissue engineering and enhancement of cellular differentiation processes has been demonstrated [24,25].

In some tissue engineering applications, it is suitable that the used scaffolds degrade in a biological environment to be gradually replaced by the newly formed tissue [26]. In this context, the most used piezoelectric materials for tissue engineering applications belong to the poly(vinylidene fluoride) family [12] and do not possess biodegradability, which may hinder some applications [27]. Further, the other often used biomaterial, poly(l-lactic acid) (PLLA), shows a piezoelectric response, however with a slow degradation rate [12].

Poly(hydroxybutyrate-co-hydroxyvalerate), PHBV, is a PHB copolymer that was developed to enhance PHB toughness and processability, widening its industrial applications [28,29]. PHBV is a biocompatible, biodegradable, highly absorbent, non-toxic thermoplastic. Together, all these properties make it a promising candidate material for biomedical applications, such as the fabrication of cardiac stents [30], wound dressing [31], drug release [32] and antitumor applications [33]. More specifically in tissue engineering, PHBV is usually employed for scaffolds in bone tissue regeneration [34,35], absorbable surgical sutures [36], among others.

Additionally, owing to its piezoelectric properties (piezoelectric coefficient of 1.3 pC/N, similar to human bone [12,37]), this polymer can provide electrical stimulation through mechanical solicitation and with a suitable degradation rate for a variety of tissue engineering applications [38,39].

In addition to tissue engineering applications, PHBV has a wide range of industrial applications such as food packaging [40], cosmetics, personal care products (towels and diapers), helmets and panels for several automotive materials [41,42,43]. More recently, this polymer was also used in the scope of environmental remediation towards nitrates and chlorine removal from contaminated water [44,45].

A biodegradable magnetoelectric composite, combining piezoelectric PHBV with magnetostrictive cobalt ferrites, CoFe_2_O_4_ (CFO) has been reported [8], confirming the ability of biodegradable PHBV and magnetoelectric compound (PHBV/CFO) to be processed into different morphologies, including microspheres, films, fibers and 3D porous scaffolds, adequate for tissue engineering applications with different structural microenvironments.

As the biodegradation process occurs, it is important to keep the properties that assure the effectiveness of the scaffold after its implantation. For this reason, morphological, physicochemical and magnetic properties must be evaluated after exposure of the samples to a physiological environment for a given time.

The work herein presented reports on the degradation of pristine PHBV and PHBV/CFO composite samples, both in the form of film, fiber and porous scaffold. Degradation took place for 8 weeks in contact with simulated body fluid (SBF) with ion concentration found in human plasma, allowing to provide insights on materials characteristics upon implantation. These results will help to fill the gap in the areas of (a) PHBV’s degradation in biological media, (b) degradation behavior of the materials depending on functional microstructures and (c) in the area of magnetoelectric compounds for tissue engineering purposes [24,46], in which the degradation behavior in a simulated biological environment has never been addressed.

## 2. Experimental

### 2.1. Materials

Poly(hydroxybutyrate-co-hydroxyvalerate), PHBV, (3% HV mol/mol, molecular weight of 460.64 g mol^−1^, 99% purity) was purchased from Natureplast (IFS, France). Chloroform (molecular weight of 119.38 g mol^−1^, 99% purity) was purchased from Fischer (Porto Salvo, Portugal, C/4960/17). CFO molecular weight 234.62 g mol^−1^, reference 1510FY, 98% purity and 35–55 nm size range) was obtained from Nanoamor (Katy, TX, USA). Sodium Chloride (molecular weight of 58.55 g mol^−1^, 98% purity, reference S/3160/60) was purchased from Fisher (Porto Salvo, Portugal).

### 2.2. Sample Processing

Samples were prepared from either a 10% (w/v) polymer solution of PHBV in chloroform or a composite solution of 10% (w/v) PHBV on chloroform with 10% (w/w) CFO [8]. In the later, CFO were sonicated for 1.5 h in chloroform before polymer dissolution. The CoFe_2_O_4_ concentration was selected due to not comprising the biocompatibility [47].

Different techniques were used to produce different morphologies and structures from both polymer and composite solutions.

Polymer films were obtained by solvent-casting. The polymer/composite solution was poured over a glass substrate, spread with a blade and left at room temperature for 2 days until the solvent has evaporated. After removal from the glass substrate, mechanically robust, self-standing films were obtained.

Fibrous samples were produced by electrospinning. Fibers were electrospun with a flow rate of 2 mL h^−1^ (Syringe pump) and an electrospinning voltage of 20 kV (Matsusada AU-30P1-L power source). Fibers were collected on a flat static collector set at 20 cm between needle tip (diameter of 0.5 mm) and collector.

Porous 3D scaffolds were produced according to the solvent-casting/particulate-leaching technique. For that, 10 g of NaCl, acting as porogen agent, was placed in a Petri dish and covered with 10 mL of polymer/composite solution. Solution and porogen were homogeneously mixed, and the Petri dish was then left at room temperature until the solvent was completely evaporated. Then, the Petri dish was immersed in deionized water to dissolve/release the porogen. Water was changed daily to avoid saturation and agitated at least twice a day. The porogen leaching procedure was carried out for 3 days. Then, the 3D scaffold was removed from the water and let to dry at room temperature.

### 2.3. Degradation Assays

**Figure 1:**
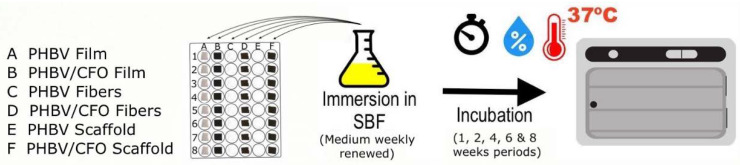
Schematic representation of the steps involved in the degradation assays.

For the degradation assays, simulated body fluid (SBF) was prepared according to [48]. Briefly, SBF was obtained by adding to 700 mL of ultrapure water under mechanical agitation the following components: NaCl (8.04 g), NaHCO_3_ (0.36 g), KCl (0.23 g), K_2_HPO_3_H_2_O (0.23 g), MgCl_2_.6H_2_O (0.31 g), HCl (39 mL), CaCl_2_ (0.29 g), Na_2_SO_4_ (0.07 g) and TRIS (6.12 g). After complete dissolution, the pH has been adjusted to 7.4 and completed the volume of 1 L. The samples from the different materials were cut in 1 cm^2^ pieces and immersed in 3 mL of SBF at 37 °C in 12-well tissue culture polystyrene plates (Figure 1).

The fluid was exchanged weekly avoiding changes in pH and ion concentration. Samples were immersed for 1, 2, 4, 6 and 8 week periods. The fluid was produced to mimic ion concentration in blood plasma, according to [48].

### 2.4. Morphological Analysis

The samples were characterized before and after the degradation assay to evaluate possible morphological changes. For that, the samples were placed in aluminum pans and sputter-coated with a thin layer of gold with a Polaron SC502 instrument (East Sussex, UK). The images of the samples were acquired on a FEG-SEM Hitachi set up with a 3 kV voltage.

### 2.5. Chemical Structure Analysis

Changes in the samples chemical structure due to the degradation process were evaluated by Fourier Transform Infrared Spectroscopy (FTIR) at room temperature with a Jasco FT/IR 4100 instrument (Jasco, Easton, MD, USA) in the Attenuated Total Reflection (ATR) mode. A total of 64 scans were used for measurement with a resolution of 4 cm^−1^ in the 400–6000 cm^−1^ range.

### 2.6. Thermal Analysis

Thermal properties were assessed by differential scanning calorimetry (DSC) with a Mettler Toledo DSC823e apparatus (Mettler Toledo, Columbus, OH, USA) at 10 °C min^−1^ heating rate in the 0–200 °C temperature range. Measurements were carried out in a nitrogen atmosphere in 40 μL aluminum pans.

### 2.7. Elemental Surface Composition Analysis

X-ray photoelectron spectroscopy (XPS) was performed to evaluate the surface elemental composition and atomic concentration of non-degraded and degraded samples in a Kratos AXIS Ultra HSA, with VISION software for data acquisition and CASAXPS software for data analysis. The experiments were carried out with a monochromatic Al Kα X-ray source (1486.7 eV), operating at 15 kV (90 W), in Fixed Analyzer Transmission (FAT) mode, with a pass energy of 40 eV for regions ROI and 80 eV for a survey. The acquisition of the data was performed with a pressure lower than 1 × 10^−6^ Pa using a charge neutralization system. The electric charge effect was corrected by the reference of the carbon peak (284.6 eV). The binding energies (BEs) were referenced to the C1s hydrocarbon peak at 286.4 eV. The resulted spectra were analyzed using the CASA XPS software (version 2.3.15, CASA Software Ltd, Teignmouth, UK). For the curve fitting of the high-resolution spectra of the samples, 30% Gaussian/70% Lorentzian mixed line shapes were used.

### 2.8. Weight Loss Assessment

Samples weight was measured before and after the contact with the degradation medium (SBF). Then, samples withdrawn from SBF medium after 1, 4, 6 and 8 weeks were dried at room temperature for at least two weeks, and the mass was measured with an XS balance BL 224 ± 0.1 mg. Three replicas were used for each of the degradation intervals.

### 2.9. Cytotoxic Assay

Indirect cytotoxicity evaluation of the different samples after 6 weeks of degradation was performed adapting the ISO 10993-5 standard test method.

MC3T3-E1 pre-osteoblast cells (Riken cell bank, Tsukuba, Japan) were cultured in 75 cm^2^ cell culture flask at 37 °C in a humidified environment and 5% CO_2_, using Dulbecco’s modified Eagle’s medium (DMEM, Biochrom, Berlin, Germany) containing 1 g L^−1^ glucose, 10% fetal bovine serum (FBS, Biochrom, Berlin, Germany) and 1% (v/v) penicillin/streptomycin solution (P/S, *Biochrom*). The sterilization of the samples was carried out by exposition to ultraviolet radiation for 1 h each side of the samples and washing with sterile phosphate-buffered saline solution (PBS, pH 7.4). Then, a suspension of 2 × 10^4^ cell mL^−1^ was seeded in 96-well tissue culture polystyrene plates and incubated for 24 h at the same conditions described above to ensure cell attachment on the plate. Simultaneously, each sample was incubated for 24 h in a 24-well tissue culture polystyrene plate. After the incubation time, the cell culture medium in the 96-well plates was removed, and 100 µL of culture medium (that was in contact with the different samples) was added to each well. Metabolic activity was then evaluated after 72 h of incubation using the 3-(4,5-dimethylthiazol-2-yl)-2,5-diphenyltetrazolium bromide (MTT) proliferation assay according to the manufacturer’s instructions (Sigma-Aldrich, Sintra, Portugal).

Briefly, the medium of every well was removed, and fresh medium containing MTT solution (5 mg mL^−1^ of MTT dissolved in DMEM in a 1:10 ratio) was added to the cells and incubated for 2 h at 37 °C in the dark. Then, the MTT solution was removed, and the precipitated formazan was dissolved with 100 µL dimethyl sulfoxide (DMSO)/well followed by measuring the optical density at 570 nm. All quantitative results were obtained from four replicate samples and presented as the average of viability ± standard deviation. The percentage of metabolic activity was calculated according to the equation reported in [49], where:

Metabolic activity (%)=(Absorbance of sample/Absorbance of negative control)×100

## 3. Results and Discussion

### 3.1. Samples Macrostructure

**Figure 2:**
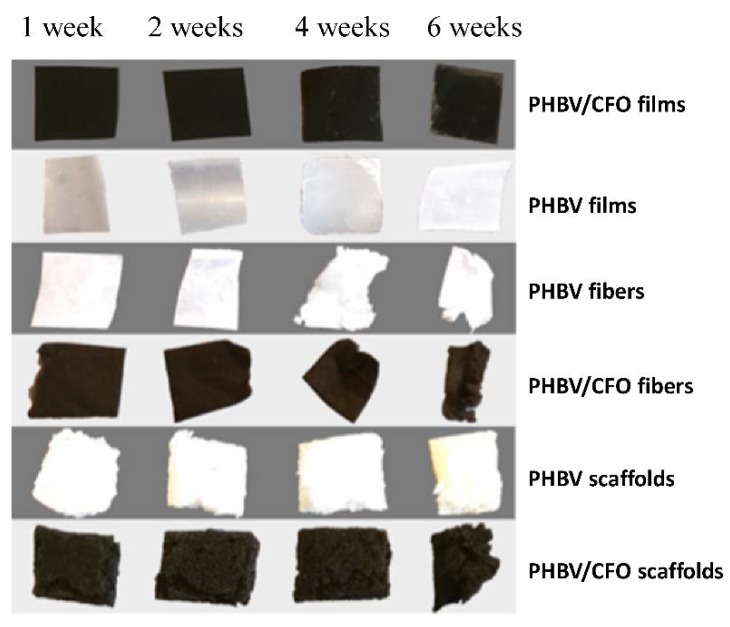
Poly(3-hydroxybutyrate-co-hydroxyvalerate) (PHBV) and PHBV/cobalt ferrite (CFO) films, scaffolds and fibers after 1, 2, 4 and 6 weeks immersion in simulated body fluid (SBF) at 37 °C.

Figure 2 shows the PHBV and PHBV/CFO samples along the degradation process (the samples before degradation look similar to the ones in week 1). Significant changes are observed in the samples, in particular in the ones with fiber morphology, after four weeks of degradation.

This visual assessment indicates that the samples with higher surface area exhibit a more advanced state of degradation after a given time, which is consistent with the degradation process of PHBV, which is caused by hydrolyzation of the polymers water solvable portions [50]. Thus, a higher surface area offers a larger interaction of the polymer with water.

After 8 weeks, severe degradation is observed in fibers and scaffolds, yielding the smallest samples residues (results not shown). On the contrary, both films showed little degradation and maintain a more pristine look throughout the assay, as verified in Figure 2. In this sense, all the morphological and physical-chemical properties were evaluated for the degraded samples after 6 weeks of immersion in SBF medium.

### 3.2. Samples Microstructure

**Figure 3:**
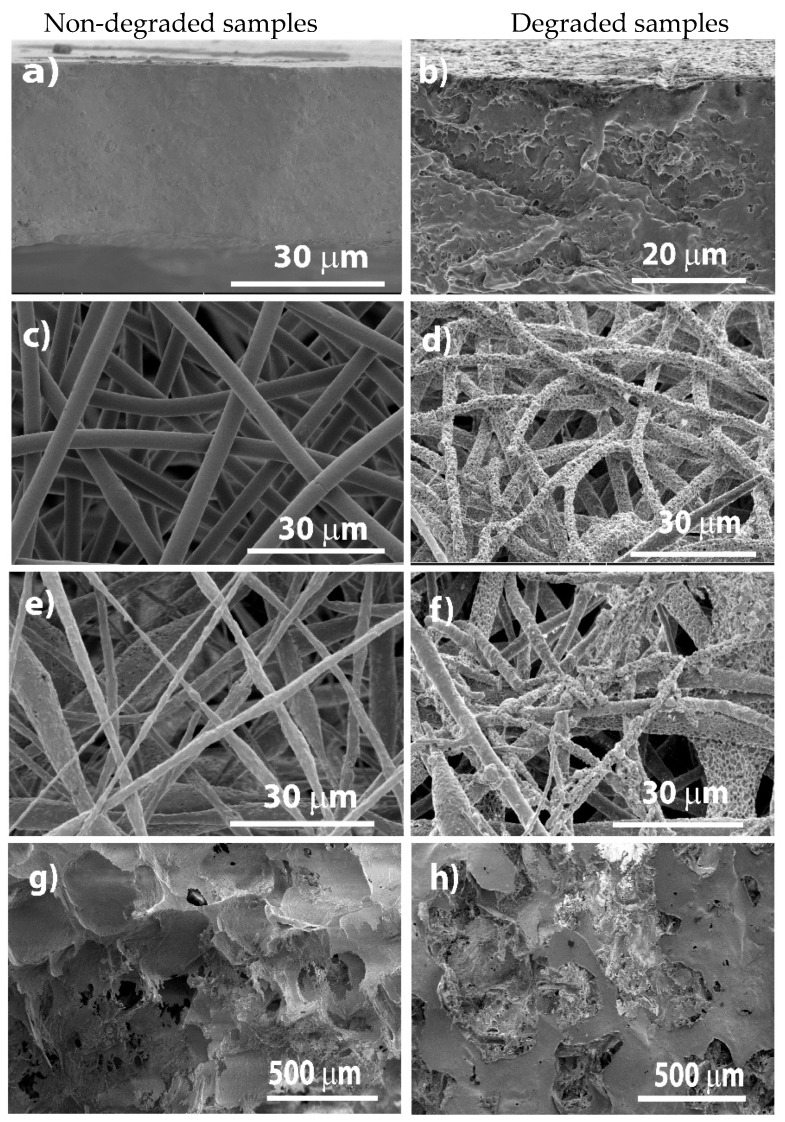
Cross-section SEM images of representative samples: PHBV/CFO films (**a**,**b**), PHBV fibers (**c**,**d**), PHBV/CFO fibers (**e**,**f**) and PHBV/CFO scaffolds (**g**,**h**), before and after degradation, respectively.

The morphology of all films and electrospun fibers before and after the degradation process was analyzed by SEM (Figure 3), where the degradation is best noticed for all the samples at week 6.

PHBV/CFO composite films (Figure 3a) reveal a homogeneous and non-porous surface, showing that the CFO particles are incorporated into the PHBV polymer matrix. The electrospun PHBV and PHBV/CFO fibers (Figure 3c,e, respectively) are characterized by a rather smooth, in particular for the pristine polymer- and high surface area with a large porous network. The fibers surface area increases due to the CFO incorporation into the PHBV matrix [8], as also confirmed by the increase in surface roughness and irregularities observed in Figure 3e for the composite sample in comparison with Figure 3c for the pristine polymer.

After 6 weeks of degradation, a significant erosion of both pristine (results not shown) and composite films occurs, increasing the surface roughness and being possible to observe the CFO particles within the PHBV matrix (Figure 3b). The erosion is homogenous all along the samples and similar for both pristine and composite materials, i.e., the presence of CFO nanofillers does not seem to influence the degradation process.

Relatively to the degradation of electrospun PHBV and PHBV/CFO fibers, Figure 3f,h, after 6 weeks of degradation, the composite fibers exhibit a rough and porous surface, while the pristine ones reveal a smooth surface. Similarly, to the composite films, some round structures can be observed in Figure 3h which represent CFO agglomerates exposed after the polymer degradation.

Comparing the different morphologies, it is verified a faster/advanced degradation state in fibers and scaffolds when compared to films, as observed in Figure 2. This is explained, as previously mentioned, by the larger surface area of fibers and scaffolds exposed to the degradation medium, allowing increased contact between the solution and the polymer.

### 3.3. Chemical Analysis

**Figure 4:**
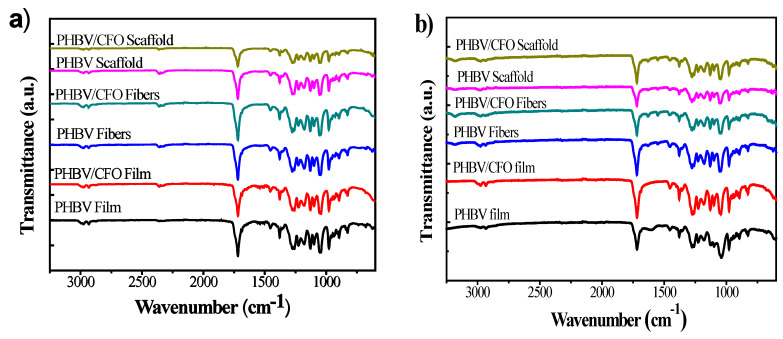

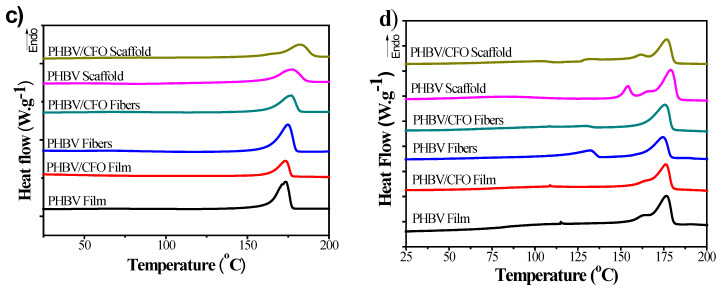
FTIR spectra of the different PHBV and PHBV/CFO morphologies before (**a**) and after (**b**) 6 weeks of immersion in SBF. DSC thermograms of pristine and PHBV/CFO composites (**c**) before and (**d**) after 6 weeks of immersion in SBF, respectively.

FTIR spectra of the samples before and after six weeks of degradation are shown in Figure 4. It is observed that no differences occur after the degradation process: the same characteristic absorption bands are observed for the degraded samples as for the PHBV pristine morphologies. Thus, the absorption bands at 826–979 cm^−1^ and 1227–1478 cm^−1^ corresponding to C-H vibrations, and at 1057 cm^−1^, 1133 cm^−1^ and 1183 cm^−1^ corresponding to C-O vibrations, are present in both pristine and composite samples [51]. The absorption bands at 2934 cm^−1^ are attributed to the C-H groups stretching. Additionally, independently of the morphology, Figure 3a,b, show no differences for the different PHBV/CFO samples, indicating that there is no chemical interaction between the polymer and CFO nanoparticles [8]. In this sense, both the morphological variations and the inclusion of CFO into the PHBV matrix does not promote changes in the molecular structure or affects the degradation mechanism of the polymer (Figure 4a,b). Figure 4b also shows a general decrease in the absorption bands intensity for both PHBV and PHBV/CFO composites, being more evidenced in fibers and scaffolds as a result of the degradation process, which is an indication of the scission of the PHBV bonds linkage.

### 3.4. Thermal Analysis

DSC analysis was performed on pristine and degraded samples to evaluate the effect of the degradation process in the thermal properties of the samples (Figure 4c,d). Independently of the sample morphology, both for pristine and PHBV/CFO composites no major changes were verified between the pristine samples (Figure 4c), being characterized by a melting process with melting temperature (T_m_) values around 160 to 180 °C. For pristine degraded PHBV films, fibers and scaffolds, peaks are observed at 161, 131 and 154 °C, respectively, (Figure 4d) indicating that the degradation process of the samples leads to a crystalline phase destabilization, starting the samples fusion at lower temperatures. Further, with the exception of the PHBV/CFO scaffolds, and as stated in [8], neither processing nor the addition of 10% CFO leads to any relevant modifications of the thermal characteristics of the samples. Similarly, after six weeks of degradation in SBF, the thermal properties of the different samples were not altered (Figure 4d). The T_m_, ΔH_m_ and crystallinity degree (X_c_) values of the different non-degraded and degraded PHBV and PHBV/CFO samples are shown in Table 1.

From the enthalpy of the melting peak, the degree of crystallinity (X_C_) of the PHBV and PHBV/CFO samples with the different morphologies was obtained by applying Equation (1).

(1) $$X_{c}=\frac{{\Delta H}_{m}}{{\Delta H}_{m100}}$$

where X_c_ is the degree of crystallinity, ∆H represents the area of the melting peaks, ∆H_m_ the enthalpy of the PHBV samples and ΔH_m100_ the enthalpy of 100% crystalline PHBV (146.6 J mol^−1^) [52].

Taking into account the crystallinity degree values observed in a previous study for the different PHBV and PHBV/CFO morphologies [8], independently of the morphology, all the degraded samples show a decrease in the degree of crystallinity. It is important to mention that in fact there are differences in the degree of crystallinity of the different non-degraded samples, as reported and discussed in [8]. These differences are entirely related to the different processing methods, in which important differences in solvent evaporation conditions and crystallization dynamics are involved, main parameters affecting the degree of crystallinity of the obtained samples. Regarding the degraded samples, the highest value of crystallinity being observed for pristine films and fibers, decreasing the crystallinity degree from 56 to 27% and 67 to 33%, respectively [8]. This decrease in the amount of crystallinity for all samples, when compared to the non-degraded samples, indicates a swelling effect of the amorphous part of the samples during the degradation process and degradation of the crystalline phase of the polymer. Further, the swelling effect is more effective for the samples that initially present higher crystallinity.

### 3.5. Elemental Surface Composition Analysis

**Figure 5:**
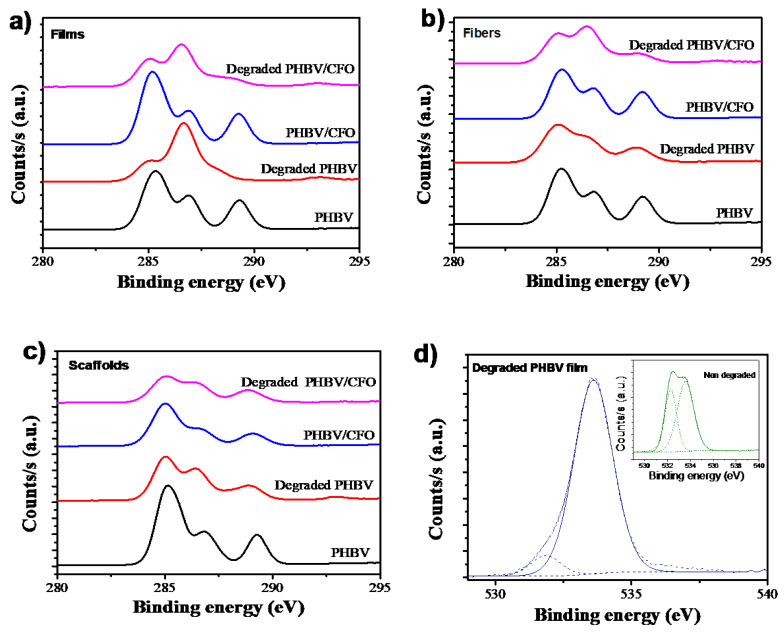
XPS results of non-degraded and degraded PHBV and PHBV/CFO samples with different morphologies: (**a**–**c**) C1s scan spectra for films, fibers and scaffolds, respectively and (**d**) O1s spectra.

XPS analysis was performed to study the chemical degradation of PHBV and PHBV/CFO samples Figure 5.

The atomic surface composition was evaluated from C1s and O1s scanning spectra. Independently of the PHBV morphology, for all the non-degraded PHBV and PHBV/CFO samples, the C1s spectra presents three peaks at 288.2 eV, 286.2 eV and 285.1 eV, attributed to the aliphatic –C=O, –C–O–C– and –C–C–/–C–H bonds of PHBV, respectively [53,54]. After the hydrolytic degradation, a decrease in the intensity of the peak at 288.2 eV related to the -C=O bond is observed. In addition, the intensity of the peaks at 286.2 eV and 285.1 eV slightly decreases with the degradation process (Figure 5a–c). These results are indicative that the hydrolytic degradation induces the breakdown of the PHBV bonds. It is also to notice that pristine and PHBV/CFO composite fibers and scaffolds present the highest decrease in the peak intensity, showing that the sample morphology influences the PHBV degradation. No significant changes are observed between the pristine and composite samples.

Figure 5d shows the representative O1s spectra for non-degraded and degraded PHBV films. Similar results are observed for the other PHBV and PHBV/CFO morphologies. For the non-degraded films, two peaks 531 and 532 eV are observed, corresponding to the aliphatic -C=O and -C-O bonds [53,54,55]. After the PHBV degradation, all samples (neat and PHBV/CFO composites) show just a single peak, which is indicative of the -C=O breakdown. The elemental composition of the samples is presented in Table 2.

**Figure 6:**
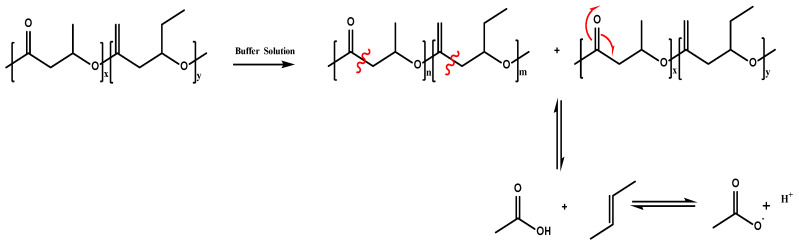
Schematic representation of the PHBV hydrolytic degradation [56].

From the elemental compositions summarized in Table 2, it is observed that the PHBV and PHBV/CFO non-degraded surface of the films is composed of ~70% of carbon and 30% of oxygen. Similar results are observed for the non-degraded fibers and scaffolds. After the degradation process, as it is also observed in the XPS spectra, clear alterations occur in the elemental composition of the samples. As shown in Table 2, the amount of carbon decreases for the PHBV and PHBV/CFO degraded samples and the amount of oxygen increases, increasing the O/C atom ratio. This fact is an indication that, during the hydrolytic degradation, the C-C and C-H bonds scission occurs preferentially due to the strength of the C=O (749 kJ mol^−1^) and C-O (360 kJ mol^−1^) bonds, as schematically illustrated in Figure 6. Further, attending to the results presented in Figure 5 and Table 2, and the schematic representation of the degradation mechanism in Figure 6, it is also possible to conclude that the presence of CFO into the PHBV polymer matrix does not influence significantly the PHBV degradation process, as, independently of the morphology, the incorporation of CFO does not promote relevant changes in the elemental composition of the samples (Table 2).

### 3.6. Weight Loss

**Figure 7:**
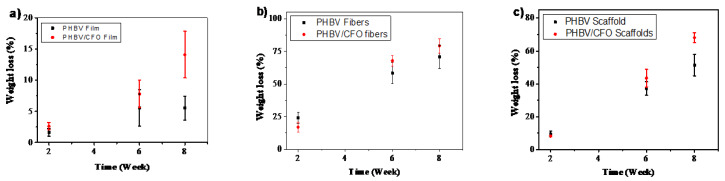
Weight loss relative to the original mass after degradation for 2, 6 and 8 weeks in SBF for (**a**) PHBV and PHBV/CFO films, (**b**) fibers and (**c**) scaffolds.

The weight loss as a function of time for the different samples morphology is shown in Figure 7. As observed, the weight loss is independent of the samples morphology, occurring along the different weeks. For pristine samples, and due to the high surface area of the fibers and scaffolds, which promotes a higher contact with the SBF medium, the highest weight loss is observed for those morphologies. After two weeks, pristine films reveal a weigh loss of ~2%, increasing the weight loss to ~5.5% in week 6 and 8. For PHBV fibers and scaffolds, the weight loss increase from 24% (week 2) to 70% and from 9% to 51%, respectively (week 8), which is in agreement with the results shown in Figure 2.

Similarly, also for the PHBV/CFO composites, fibers and scaffolds reveal the highest weight loss along time due to the high porous structure of these morphologies when compared with the dense and compact films. Further, Figure 7 also shows that the inclusion of CFO particles into the PHBV matrix induces a slight faster weight loss when compared with the pristine ones.

### 3.7. Cytotoxic Analysis

**Figure 8:**
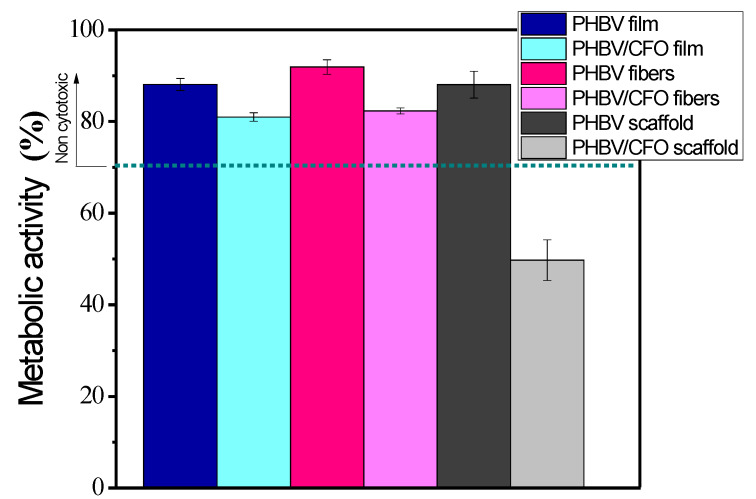
Cytotoxicity assay results of MC3T3-E1 pre-osteoblast cells in contact with the as-prepared extraction media exposed to the different PHBV samples after six weeks of degradation for 72 h (relative metabolic activity was presented as the percentage of the negative control with n = 4 ± Standard Deviation).

The PHBV and PHBV/CFO samples produced with different morphologies show no cytotoxic behavior [8], proving for the composite samples that the CFO is fully incorporated into the polymer matrix. Degradation can lead to cytotoxic effects either due to the exposure of specific polymer groups or to the presence of exposed CFO. Thus, the samples obtained after six weeks of degradation were submitted to a cytotoxic assay, and the metabolic activity is presented in Figure 8.

Figure 8 shows that all samples remain non cytotoxic (metabolic activity values higher than 70%, according to the ISO 10993-5) except for the PHBV/CFO scaffold, which is the one of the samples with the larger exposed surface area and with encapsulated CFO nanoparticles. In this way, it is confirmed that the degradation of PHBV does not lead to the formation of cytotoxic components. On the other hand, for composites samples, and once CFO can show cytotoxic effects [57], it is possible to infer that the particles continue efficiently encapsulated, excepting in the case of the scaffold morphology, as it suffers the largest degradation among the different samples. Therefore, an alternative to CFO, such as an iron oxide (Fe_3_O_4_), with lower magnetization and magnetostriction, but biocompatible, can be used for tissue engineering applications when the piezoelectric polymer is expected to be degraded.

## 4. Conclusions

Electroactive poly(3-hydroxybutyrate-co-hydroxyvalerate) (PHBV) with and without cobalt ferrite, CFO, nanoparticles were produced in the form of films, fibers and scaffolds. These materials were placed in contact with a simulated body fluid up to 8 weeks in order to study their degradation. For this purpose, the morphology, physical, chemical and thermal properties were evaluated. It is shown that the degradation process mainly affects the morphology and the degree of crystallinity of the samples. The elemental surface composition analysis shows that the hydrolytic degradation induces the breakdown of the PHBV bonds, not affecting the cytotoxic behavior of PHBV and PHBV/CFO samples, which are not cytotoxic, except the PHBV/CFO scaffold, due to the exposition of the CFO fillers after the degradation of the polymer. Hence, the proposed systems show suitability for magneto and electro-active tissue engineering applications, but magnetic fillers such as biocompatible Fe_3_O_4_, must be used when the piezoelectric materials are expected/needed to be degraded and the magnetic fillers exposed or released.

## Figures and Tables

**Table 1:** T_m_, ΔH_m_ and X_C_ of PHBV and PHBV/CFO composite non-degraded [8] and degraded samples. The associated error is ±2%.

	Sample	T_m_ (°C)	ΔH_m_ (J/g)	X_C_ (%)
**Films**	PHBV	174	82	56
	PHBV/CFO	177	70	48
	PHBV (Degraded)	173	40	27
	PHBV/CFO (Degraded)	173	66	45
**Fibers**	PHBV	175	98	67
	PHBV/CFO	177	67	46
	PHBV (Degraded)	171	49	33
	PHBV/CFO (Degraded)	172	66	45
**Scaffolds**	PHBV (Degraded)	176	45	31
	PHBV/CFO (Degraded)	173	37	25

**Table 2:** Surface chemical composition of non-degraded and degraded PHBV and PHBV/CFO samples.

	Samples	Elemental Composition (%)		
		C	O	O/C
**Non-degraded**	PHBV film	69.4	30.6	0.44
	PHBV/CFO film	71.9	28.1	0.39
	PHBV fibers	70.3	29.7	0.42
	PHBV/CFO fibers	69.0	31.0	0.45
	PHBV scaffolds	73.2	26.8	0.37
	PHBV/CFO scaffolds	73.3	26.7	0.36
**Degraded**	PHBV film	66.8	33.3	0.49
	PHBV/CFO film	63.7	36.0	0.56
	PHBV fibers	69.3	30.7	0.44
	PHBV/CFO fibers	66.5	33.4	0.50
	PHBV scaffolds	63.2	36.8	0.58
	PHBV/CFO scaffolds	68.3	31.7	0.46
